# Dopaminergic Inhibition of the Inwardly Rectifying Potassium Current in Direct Pathway Medium Spiny Neurons in Normal and Parkinsonian Striatum

**DOI:** 10.3390/brainsci15090979

**Published:** 2025-09-12

**Authors:** Qian Wang, Yuhan Wang, Francesca-Fang Liao, Fu-Ming Zhou

**Affiliations:** Department of Pharmacology, Addiction Science and Toxicology, College of Medicine, University of Tennessee Health Science Center, Memphis, TN 38103, USA

**Keywords:** basal ganglia, cognition, dopamine receptor, inwardly rectifying potassium channel, medium spiny neuron, Parkinson’s disease, striatum

## Abstract

**Background:** Despite the profound behavioral effects of the striatal dopamine (DA) activity and the inwardly rectifying potassium channel (Kir) being a key determinant of striatal medium spiny neuron (MSN) activity that strongly affects behavior, previously reported DA regulations of Kir are conflicting and incompatible with MSN function in behavior. **Methods and Results:** Here, we used DA depletion mouse models that have hyperfunctional DA receptors such that potential DA regulation of Kir may be enhanced and relatively large and thus detected reliably. We show that in striatal brain slices from normal mice with an intact striatal DA system, the predominant effect of DA activation of D1Rs in D1-MSNs is to cause a modest depolarization and an increase in input resistance by inhibiting Kir, thus moderately increasing the spike outputs from behavior-promoting D1-MSNs. In brain slices from parkinsonian (DA-depleted) striatum, DA increases D1-MSN intrinsic excitability more strongly than in normal striatum, consequently more strongly increasing D1-MSN spike firing that is behavior-promoting. This DA inhibition of Kir is occluded by the Kir blocker barium chloride (BaCl_2_). In behaving parkinsonian mice, BaCl_2_ microinjection into the dorsal striatum stimulates movement and also occludes the motor stimulation of D1R agonism. **Conclusions:** Taken together, our results resolve the long-standing question about what D1R agonism does to D1-MSN excitability in normal and parkinsonian striatum and strongly indicate that D1R inhibition of Kir is a key ion channel mechanism that mediates the profound motoric and behavioral stimulation of striatal D1R activation in normal and parkinsonian animals.

## 1. Introduction

Dopaminergic (DA) activity is highly concentrated in the striatum with an intense DA innervation and a high level of segregated expression of DA D1 and D2 receptors in direct pathway medium spiny neurons (dMSNs or D1-MSNs) and indirect pathway medium spiny neurons (iMSNs or D2-MSNs) [1,2]. This DA activity is absolutely required for normal motor function: Loss of the striatal DA or blockade of striatal DA receptors leads to loss of motor function in both humans and animals; DA and D1R-like (D1R hereafter) agonists profoundly stimulate motor activity [3,4,5,6]. However, the ion channel and cellular mechanisms underlying DA’s spectacular motor stimulation are poorly understood with conflicting results in the literature that cannot explain dopaminergic motor stimulation [7,8,9,10]. D1R-bypassing optogenetic activation of the D1R-expressing medium spiny neurons (D1-MSNs) promotes motor function [11,12], but critical and long-standing questions remain: How does D1R agonism (the endogenous mechanisms) affect D1-MSN intrinsic excitability that can affect the spiking activity of these neurons? What ion channels are critically involved, and what is the key ion channel that mediates DA-D1R agonistic motor stimulation? Delineating these fundamental mechanisms will advance our understanding of basal ganglia physiology and the pathophysiology and treatment of PD and other DA and striatum-dependent neurological and psychiatric diseases.

Because the inwardly rectifying potassium channel (Kir; molecularly Kir2 type, mainly Kir2.3 subtype in MSNs) is highly expressed and tonically active in MSNs and a key determinant of MSN’s low intrinsic excitability (low input resistance and very negative resting membrane potential) [13,14,15,16,17], Kir is a key candidate for DA regulation of MSN activity. Multiple studies have investigated how D1R agonism affects MSN intrinsic excitability and reported that D1R agonism caused hyperpolarization and increased the Kir-mediated inward rectification and hence decreased intrinsic excitability and spike firing in MSNs in dorsal striatum (DS) and nucleus accumbens (NAc) [9,18,19,20]. But these results were confounded because recordings were made in mixed, unidentified D1- and D2-MSNs, and D1Rs are expressed only in 50% of the MSNs; thus, it is impossible to have a true D1R response in all recorded MSNs as reported [19]. A more recent study in the identified D1-MSN and D2-MSNs reported that D1R agonism enhanced Kir and decreased D1-MSN excitability, and D2R agonism did the opposite in D2-MSNs [10]. However, these reported DA effects are incompatible with the function of these MSNs in animal behavior: optogenetic activation of D1-MSNs stimulates motor behavior [11,12,21], and striatal D1 agonist microinjection stimulates movement [22]. Thus, the reported D1R agonistic enhancement of Kir in D1-MSNs would inhibit behavior, contradicting the easily and reliably observed behavioral stimulation of striatal D1R agonism. A more recent study [23] also did not resolve the question, as discussed in the Discussion section.

Additionally, previous studies have reported contradictory effects of DA loss on intrinsic membrane properties such as resting membrane potential (RMP) and input resistance (R_In_) measured in the same cell type in the same animal model. For example, Lieberman et al. (2018) [24] reported that D1-MSNs had more depolarized RMP and higher R_In_ in Pitx3Null mice than in wild-type (WT) mice, but Suarez et al. (2018) [25] reported that D1-MSNs had similar RMP and R_In_ in Pitx3Null mice and WT mice. It is no wonder that Humphries and Prescott lamented that the reported DA effects on striatal MSNs have been “in a state of permanent controversy” [7]. Thus, new studies are needed to determine the true DA effects in D1-MSNs and the key underlying ion channel mechanism that contribute critically to the profound motoric and behavioral stimulation of striatal D1R activity.

## 2. Materials and Methods

### 2.1. Animals

**Figure 1:**
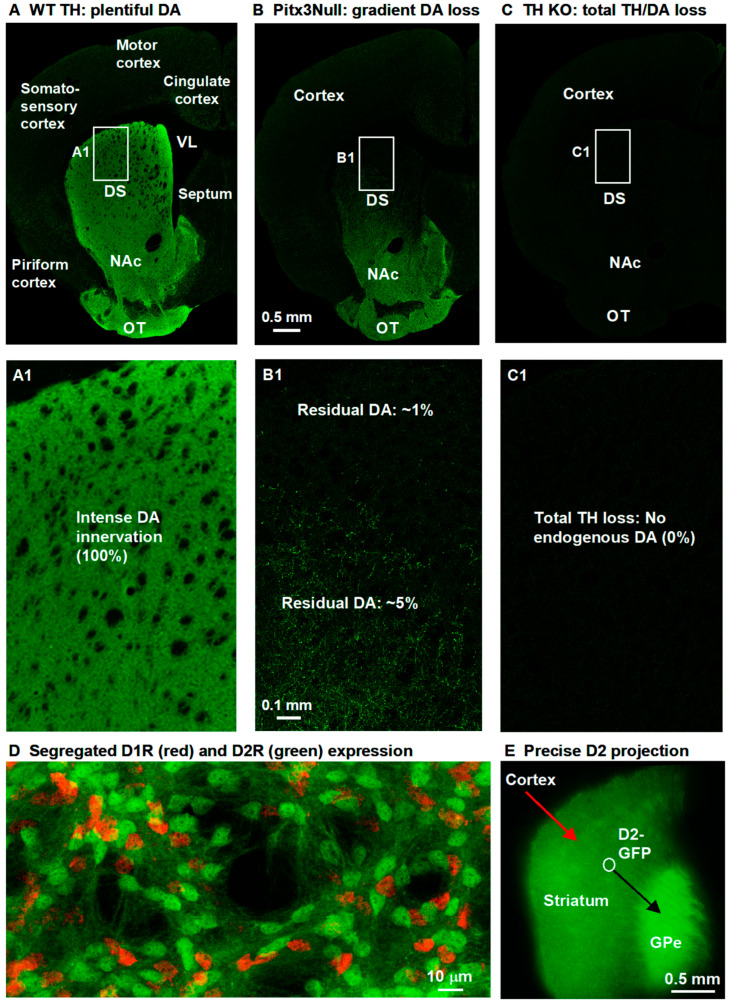
Different DA denervation patterns of Pitx3Null mice and TH KO mice. (**A**) Normal WT mice have an intense DA innervation in the entire striatum, as indicated by the intense TH immunostaining signal. (**B**) Pitx3Null mice have a dorsal–ventral DA loss gradient in the striatum (~50% residual DA in NAc). (**C**) TH KO mice have a total lack of TH in the entire striatum. The boxed areas are expanded and displayed below the main images. Severe DA loss leads to hyperfunctional DA receptors in the dorsal striatum in Pitx3Null mice and in the entire striatum in TH KO mice, as indicated by L-dopa-treatment-induced pERK and cFos expression (see data in our prior publications: Figure 1 of Ding et al., 2015 [26] and Figure 1 of Zhong et al., 2023 [32]). (**D**) shows the completely segregated D1R and D2R expression in the two types of MSNs: direct pathway MSNs (dMSNs or D1-MSNs) and indirect pathway MSNs (iMSNs or D2-MSNs). The partial overlap of some red and green neurons is due to 2D projection. (**E**) shows that GFP neurons project to GPe only, further validating GFP (indicating D2R) is segregated in D2-MSNs only.

**Transcription factor Pitx3-/- null mutant (Pitx3Null) mice.** We have used this spontaneous mutant mouse line extensively and have a deep understanding about this mouse [4,22,26,27,28,29,30,31,32]. Pitx3 is required for the survival of most DA neurons in the substantia nigra, whereas about 50% DA neurons in the ventral tegmental area do not require Pitx3 to survive; hence, these mice have a severe and selective DA neuron loss in the substantia nigra and a severe DA denervation in the dorsal striatum, resembling the DA loss pattern in the PD brain (Figure 1A,B) [4,22,26,27,28,29,30,31,32,33,34]. In our prior studies, we observed no sex difference in DA denervation and response to L-dopa in Pitx3Null mice.

Two breeding pairs of heterozygous Pitx3+/− mice (stock # 000942) were purchased from the Jackson Laboratory (Bar Harbor, ME, USA), resulting in a small colony of homozygous Pitx3−/− (Pitx3Null), heterozygous Pitx3+/−, and wild-type Pitx3+/+ (Pitx3WT) mice at the animal facility at UTHSC in Memphis, TN, USA. Pitx3Null mice are aphakic and thus clearly identified [30,33]. The genotypes were also determined by PCR-based genotyping to identify WT, homozygotes, and heterozygotes [27]. Pitx3Null mice can survive and reproduce without any treatment, clearly due to the residual DA neurons in the midbrain and residual DA innervation in the striatum (Figure 1B). D1Rs and D2Rs in the dorsal striatum in Pitx3Null mice are also hyperfunctional due to DA denervation in the dorsal striatum (Figure 1B) [22,31,32,34]. We detected striatal DA receptor hyperfunctionality (indicted by L-dopa-induced pERK and cFos expression) in Pitx3Null mice as early as PN 10, the youngest we tested (unpublished data of Zhou lab; data from PN14 were published [32]). In parallel with DA receptor hyperfunctionality, L-dopa or D1 agonist SKF81297 stimulated motor activity in Pitx3Null mice starting at a young age (the youngest age we tested was PN10: unpublished data of Zhou lab); the dopaminergic motor stimulation in Pitx3Null mice were robust by PN18-20 and became more robust by PN30 (for example, because of increasing DA receptor expression during this period); our observations are consistent with the facts that Pitx3Null mice at PN40 were used to characterize dopaminergic motor stimulation [35] and that L-dopa stimulates motor activity in human infants/young children with genetic DA deficiency, resembling dopaminergic motor stimulation in old people with Parkinson’s disease [36].

**DA neuron-selective tyrosine hydroxylase (TH) KO mice with hyperfunctional DA receptors.** In these mice, the **TH** gene in DA neurons is selectively deleted [37]. Thus, there is a total lack of TH and DA in the entire striatum (Figure 1A,C), but the DA axons are intact and can make DA from L-dopa. An established feature of these mice is their hyperfunctional D1Rs and D2Rs [32,38,39]. TH KO mice require daily L-dopa treatment to have some motor function for eating, drinking, copulation, and surviving. The dopaminergic motor stimulation in TH KO mice, starting around PN14, was extensively characterized by the Palmiter lab, the creator of TH KO mice [37,38,40]. We observed dopaminergic motor stimulation in PN10 TH KO mice (the youngest age we tested).

Breeders for these mice was generously donated by Dr. Martin Darvas, University of Washington [41]. This mouse is now also available at the Jackson Laboratory: Stock # 009688 [B6;129-Dbh^tm2(Th)Rpa^ Th^tm1Rpa^/J]. TH KO mice are identified by PCR-based genotyping and by their baseline akinesia and their robust motor response to IP injected L-dopa.

Additionally, two pairs of heterozygous BAC D2-GFP+/− breeder mice [42] were purchased from the Mutant Mouse Research Resource Centers (MMRRC) ([stock Tg(Drd2-EGFP) S118Gsat/Mmnc (000230-UNC); RRID: MMRRC_000230-UNC]). The D2-GFP labeling is robust and highly selective (Figure 1D,E), as reported by the creator [42] and other scientists [43]. D2-GFP mice were crossed to TH KO mice and Pitx3Null mice, eventually producing TH KO-D2-GFP mice and Pitx3Null-D2-GFP mice, enabling recording identified MSNs in brain slices. Mice had free access to food and water. All animal procedures were approved by the Institutional Animal Care and Use Committee of the University of Tennessee Health Science Center, Memphis, TN, USA (protocol #20-0146, approved on 14 May 2020; protocol #23-0438, approved on 15 May 2023).

### 2.2. Patch Clamp Electrophysiology

**Brain slice preparation.** Coronal brain slices containing the striatum were prepared using a conventional method described before [30,31,44]. Mice of postnatal (PN) days 18–20 were used for the majority of our experiments; PN28-30 mice were also used to verify that conclusions from PN20 mice are still correct at a more mature age. These mice were euthanized by decapitation, and their brains were dissected out quickly and immediately immersed in an oxygenated ice-cold cutting solution (containing, in mM, 220 glycerol, 2.5 KCl, 1.25 NaH_2_PO_4_, 25 NaHCO_3_, 0.5 CaCl_2_, 7 MgCl_2_, and 20 D-glucose) for 2 min. Three hundred-micrometer-thick brain slices were cut on a Leica Zero Z VT1200S vibratome (Leica Microsystems, Wetzlar, Germany). The slices were transferred to a holding chamber filled with a standard extracellular bathing solution (containing, in mM, 125 NaCl, 2.5 KCl, 25 NaHCO_3_, 1.25 NaH_2_PO_4_, 2.5 CaCl_2_, 1.3 MgCl_2_, and 10 D-glucose) that was continuously bubbled with 95% O_2_-5% CO_2_ at 34 °C for 30 min (this solution was also used for current clamp recording), and then the holding chamber was kept at room temperature (25 °C). Brain slices were used within 1–6 h after being prepared. Ascorbic acid (vitamin C, 0.4 mM) was added to all solutions to protect the brain tissue.

**Brain slice-patch clamp recording.** Brain slices were placed in a recording chamber and continuously perfused at 2 mL/min with the standard extracellular bathing solution saturated with 95% O_2_-5% CO_2_. Recordings were made under visual guidance of a video microscope (Olympus BX51WI and Zeiss Axiocam MRm digital camera) equipped with Nomarski optics and a 60X NA 0.9 water immersion lens. D1-MSNs were identified as D2-GFP negative cells in transgenic D2-eGFP mice. This identification is reliable based on the fact that D1Rs and D2Rs are overwhelmingly if not completely segregated, especially in DS and NAc, the striatal areas this study targeted [43] (see also Figure 1D,E). Our prior study further indicated that D1-MSNs so identified have a D1R-mediated presynaptic facilitation whereas D2-MSNs have a D2R-mediated presynaptic inhibition [31]. Cholinergic interneurons are characteristically large neurons and easily avoided; GABAergic interneurons are significantly larger than MSNs, can be recognized by trained eyes, and have membrane properties distinct from those of MSNs [13,31,45,46,47]. Thus, our identification of D1-MSNs was reliable.

A Multiclamp 700B amplifier, pClamp 9.2 software, and Digidata 1322A interface (Molecular Devices, Sunnyvale, CA, USA) were used to acquire data. Patch pipettes were pulled from glass capillary tubing (1.1-mm ID, 1.5-mm OD, purchased from Sutter instrument, Novato, CA, USA: cat. # BF150-110-10) using a PC-10 puller (Narishige, Tokyo, Japan) and had resistances of 4 MΩ for recording. All recordings were made at 30–32 °C.

The intracellular solution used for recording membrane potential and currents contained (in mM) 135 KCl, 0.5 EGTA, 10 HEPES, 2 Mg-ATP, 0.2 Na-GTP, and 4 Na_2_-phosphocreatine with pH 7.25 and 285 mOsm. To voltage clamp-record Kir and other K currents, we used the following relatively high KCl and zero Ca^2+^ extracellular solution containing (in mM) 125 NaCl, 3.5 KCl, 25 NaHCO_3_, 1.25 NaH_2_PO_4_, 3.8 MgCl_2_, and 10 D-glucose. The calculated K reversal potential was at −87.5 mV for our extracellular and intracellular solution pair. Tetrodotoxin (TTX; 1 μM) was added to the extracellular solution for recording K currents. All recordings were made in the presence of 6,7-dinitro-quinoxaline-2,3-dione (DNQX; 10 µM), d,l-2-amino-5-phosphonovalerate (AP5; 20 µM), and 100 µM picrotoxin in extracellular bathing solution to block ionotropic glutamate receptors and GABA_A_ receptors. Our pilot experiment indicated that D2 antagonist eticlopride did not affect the DA excitation of D1-MSNs in TH KO mice, confirming that the DA effects recorded in D1-MSNs were direct effects on D1-MSNs.

Electrical signals were filtered at 10 kHz using the built-in four-pole low-pass Bessel filter in the Multiclamp 700B patch clamp amplifier and digitized at 20 kHz using Digidata 1322A and pClamp 9 software. At least five mice were used to obtain an averaged electrophysiological data point, with each mouse yielding one or two useful cells. The Clampfit 9.2 software was used to analyze current clamp and voltage clamp data for membrane potentials, input resistance (R_In_), membrane charging time constant (τ), action potentials and Kir; R_In_ and τ were measured using the −20 pA-induced hyperpolarization from the resting membrane potential near −80 mV. R_In_ and τ are membrane potential-dependent as documented in the classic study of Nisenbaum and Wilson (1995) [13]. Because DA induced only a modest depolarization of a few mV and the MSN was still at −75 mV or more negative, our R_In_ and τ values in different groups are comparable and changes can be attributed to changes in Kir.

**Extraction of Kir.** The voltage dependence or the I–V relationship of the DA-sensitive current or Kir was determined by using a linear voltage ramp ranging from −120 mV to −40 mV at a speed of 0.15 mV/ms, followed by a digital subtraction procedure (DA-sensitive K current = control current − current under DA). Because Kv currents are large and cannot be selectively blocked without inhibiting Kir and because the recording conditions before and during DA application usually cannot be absolutely identical, especially for access resistance, the subtraction-extracted difference Kir current (under voltage clamp) and its I–V become unreliable when the difference Kir is small, e.g., only 20 pA. But current-clamp recording is less sensitive to access resistance change. Thus, we focused on current-clamp experiments in normal WT animals. Certainly, this technical problem became small when the DA effects on Kir were large in parkinsonian striatum, allowing us to perform both current-clamp and voltage clamp experiments to delineate DA-induced changes in Kir in D1-MSNs. Additionally, we did not use tetraethylammonium (TEA) to block Kv channels to isolate Kir because TEA can also block the inward rectification or Kir in MSNs [13].

### 2.3. Unilateral Intrastriatal Microinjection and Contralateral Rotation

For this set of experiments, we used 4–5-month-old TH KO mice. Bilateral 26-gauge microinjection guide cannulas were implanted with the guide cannula tip being 1 mm above the target area in both sides of the dorsal striatum as we have used in our prior studies [22,41]. An individual internal cannula that is 1 mm longer than implanted guide cannulas was used to reach our target brain area, the dorsal striatum with the coordinates being AP 0.7 mm, ML ±2 mm from bregma, and DV −3.0 mm from the surface of skull. After a recovery of at least 10 days, behavioral testing was always performed in the afternoon. On the testing day, a tiny dose of L-dopa (1 mg/kg) with benserazide was given to the TH KO mouse by intraperitoneal injection 30 min before behavioral testing, such that the mouse had the physical strength to rotate when the activity and function of the two sides of the striatum became unbalanced upon unilateral drug microinjection into the striatum. The mouse was placed in a 24 cm × 24 cm square recording cage and was video-recorded, starting at 10 min before drug injection and ending at 60 min after unilateral drug microinjection. In this study, 0.2 µg BaCl_2_-2H_2_O (i.e., 0.2 µL 4 mM solution), 0.2 µg SKF81297, and a mixture of 0.2 µg BaCl_2_ and 0.2 µg SKF81297 (all in the fixed 0.2 µL saline) were slowly microinjected into a single side of the dorsal striatum at the speed of 40 nL/min, inducing unilateral rotations. As a motor behavioral readout, rotations can be easily recognized and were manually counted and binned into 10 min segments. A mouse received only one microinjection on the testing day, and an interval of at least 2 days was given to allow the mouse to recover fully before the next microinjection. Upon completion of the experiments, the mouse was euthanized, and the brain was processed for verification of cannula placement with bright field photographs taken using an Olympus IX50 microscope and a DP71 digital camera (Olympus Corporation, Tokyo, Japan).

### 2.4. Immunohistochemistry

Conventional immunofluorescence methods were used to detect DA axons in the striatum [1,4,30,32,34,48]. The brains were fixed in 4% paraformaldehyde dissolved in phosphate buffered saline (PBS) at 4 °C overnight and then sectioned on a vibratome. The free-floating sections (50 μm in thickness) were incubated with 1% normal donkey serum, 1% normal bovine serum (cat. # 017-000-121 and 001-000-121, Jackson ImmunoResearch, West Grove, PA, USA), and 0.4% Triton X-100 in PBS for 1 h at room temperature to block nonspecific binding and permeabilize the cell membrane, respectively. After thorough rinsing, the free-floating sections were incubated for 48 h at 4 °C with the primary antibody, a polyclonal tyrosine hydroxylase antibody produced in rabbit (diluted at 1:1000; cat. # NB300-109, Novus Biologicals, Littleton, CO, USA), and then rinsed (10 min × 3) in PBS, followed by incubating with a donkey anti-rabbit secondary antibody conjugated with the green Alexa Fluor 488 (diluted at 1:400; Invitrogen/ThermoFisher, Boston, USA) for 3 h at room temperature. For D1R staining, the primary D1R antibody is a rat monoclonal antibody purchased from Sigma (cat. # D-187, Sigma, St. Louis, MO, USA; used at 1:500 dilution). Donkey anti-rabbit secondary antibody conjugated with green Alexa Flour 488 and donkey anti-rat secondary antibody conjugated with green Alexa Flour 488 were purchased from Invitrogen (cat. # A21206 and cat. # A21208). Confocal microscopic images were acquired using a Zeiss 710 laser scanning confocal microscope and the associated image acquisition and processing software Zen (Carl Zeiss, Jena, Germany) at the Neuroscience Institute Imaging Center of the University of Tennessee Health Science Center, Memphis, TN, USA.

### 2.5. Drugs and Chemicals

6,7-dinitroquinoxaline-2,3-dione (DNQX) (cat. # 0189), 2-amino-5-phosphonopentanoic acid (AP5) (cat. # 0106), and picrotoxin (cat. # 1128) were purchased from Tocris. Barium chloride (BaCl2-2H2O) (cat. # 217565) was purchased from Sigma-Aldrich (St. Louis, MO, USA). Dopamine HCl (cat. # H8502) was purchased from Sigma-Aldrich. Tetrodotoxin was purchased HelloBio.com (#HB1035, Princeton, NJ, USA). SKF81297-HBr was purchased from Tocris (cat. # 1447). Routine chemicals like sodium chloride were purchased from Sigma-Aldrich (St. Louis, MO, USA).

We need to note here that because D1Rs and D2Rs are now firmly established to segregate in the 2 groups of MSNs [43] (we also have ample data for this conclusion, see Figure 1D,E); bath application of DA will activate only D1Rs in D1-MSNs and separately D2Rs in D2-MSNs in a coronal brain slice preparation with NMDA and non-NMDA glutamatergic receptors and GABA_A_ receptors blocked, but D2R activation does not affect D1-MSNs under our recording condition. We chose DA because it is the endogenous neurotransmitter for DA receptors and hence more physiologically relevant than selective exogenous DA agonists.

### 2.6. Statistical Analysis

Values were expressed as mean ± SEM. The paired Student’s t test was used to compare changes in the same groups of cells before and during drug application. The un-paired t-test was used to compare the measurements of the same parameter before and after drug treatment in the same mouse group. One-way ANOVA and post hoc Tukey test were used to compare drug-induced rotations 10 min after drug injection in TH KO mice. Following the recent professional guidance on statistics [49,50], *p* values are given in the text, the figures, or the table, but the traditional binary threshold at *p* = 0.05 and the phrases “statistically significant” and “statistically not significant” are not used; instead, we base our conclusions on the totality of the data.

## 3. Results

### 3.1. DA Modestly Increases D1-MSN Excitability in the Dorsal Striatum (DS) in Normal Mice with Intact DA Innervation

**Figure 2:**
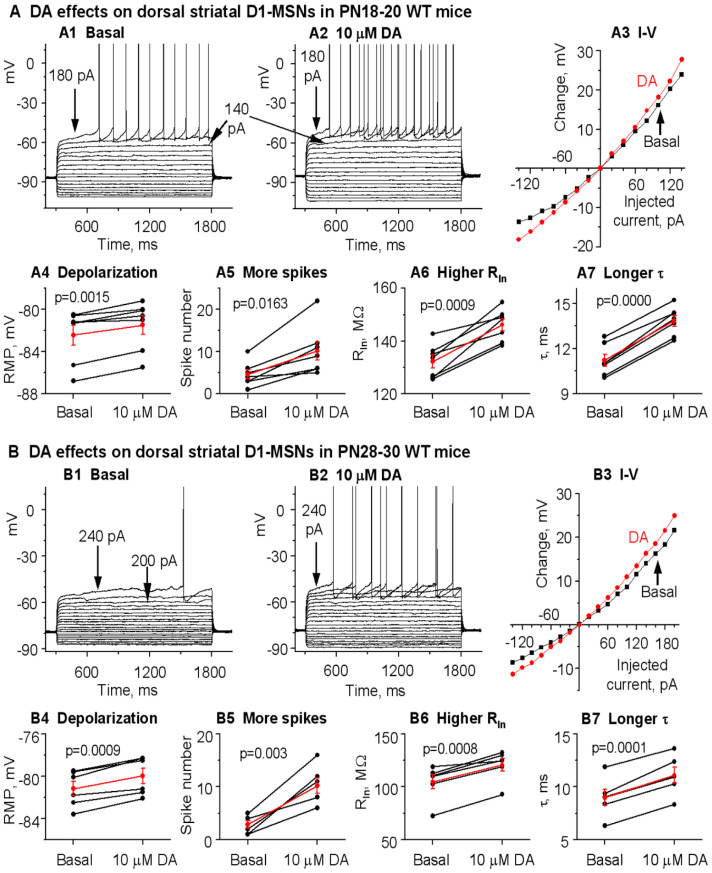
DA normally and modestly increases D1-MSN excitability in the dorsal striatum (DS) in PN18-20 and PN28-30 WT mice with intact DA innervation. **A.** An example PN20 D1-MSN receiving hyperpolarizing and depolarizing current injection before (**A1**) and during (**A2**) bath application of 10 μM DA. Current pulse-induced voltage changes are quantified in **A3**. **A4**–**A7** are pooled data showing the DA effect on RMP (**A4**), action potential firing (**A5**), R_In_ (**A6**) and τ (**A7**) in 7 D1-MSNs. p values are from paired t-tests. **B**. An example PN28 D1-MSN receiving hyperpolarizing and depolarizing current injection before (**B1**) and during (**B2**) bath application of 10 μM DA. Current pulse-induced voltage changes are quantified in **B3**. **B4**–**B7** are pooled data showing the DA effect on RMP (**B4**), action potential firing (**B5**), R_In_ (**B6**) and τ (**B7**) in 6 D1-MSNs. p values are from paired t-tests. Spikes are truncated for displaying subthreshold responses.

We first examined DA effects on D1-MSN intrinsic excitability and spike firing, in brain slices with fast glutamatergic and GABA receptors blocked, in DA innervation-intact DS and NAc in normal mice. To increase recording quality and data reliability, we started with PN18-20 mice that yield more viable brain slices and hence high-quality recordings. After obtaining a stable baseline recording in current clamp mode, 10 μM DA was bath-applied. We observed that 10 μM DA had modest but consistent excitatory effects on these D1-MSNs by causing a depolarization, increasing the whole cell input resistance (R_In_), increasing the membrane charging time constant (τ), and increasing the number of depolarizing current pulse-triggered spikes. Examples and pooled data ― scatter plots of these effects are displayed in Figure 2A and the numerical values are listed in Table 1. Bath application of 10 μM DA did not affect the threshold membrane potential for action potential firing, the action potential waveform, or the afterhyperpolarization. Similar DA effects were observed in D1-MSNs in DS in PN28-30 WT mice (Figure 2B). [Choosing PN18-20 mice and PN28-30 mice was for practical reasons: basal membrane properties and DA effects at these 2 ages are quantitatively different: Kir and DA effects were higher at PN28-30 than at PN18-20; more mature animals were not used due to low patch clamping experimental yields in mature animals.] As evident in Figure 2(A1–A3) and Figure 2(B1–B3), a consistent DA effect was a reduction in the inward rectification, and this effect became larger as the membrane potential became more negative, indicating a mediation of DA-triggered inhibition of Kir, although we were unable to obtain reliable voltage clamp I-V data because this potential DA agonism-inhibited Kir was likely to be <20 pA (more details in the Method section: Extraction of Kir).

### 3.2. DA Hyperactively Increases D1-MSN Excitability in the DS in TH KO Mice with Total DA Loss

Next, we examined how DA/D1 agonism affects the intrinsic excitability of D1-MSNs in parkinsonian TH KO mice (Figure 1C) in which DA (derived from injected L-dopa) or D1R agonism produces profound motor stimulation [38]. We first performed current-clamp experiments followed by current-clamp experiments; these two types of experiments not only provide independent information on neuronal excitability but also verify each other, thus increasing data reliability. Again, to increase recording quality and data reliability, we started with PN18-20 mice that yield more viable brain slices and hence high quality recordings.

**Figure 3:**
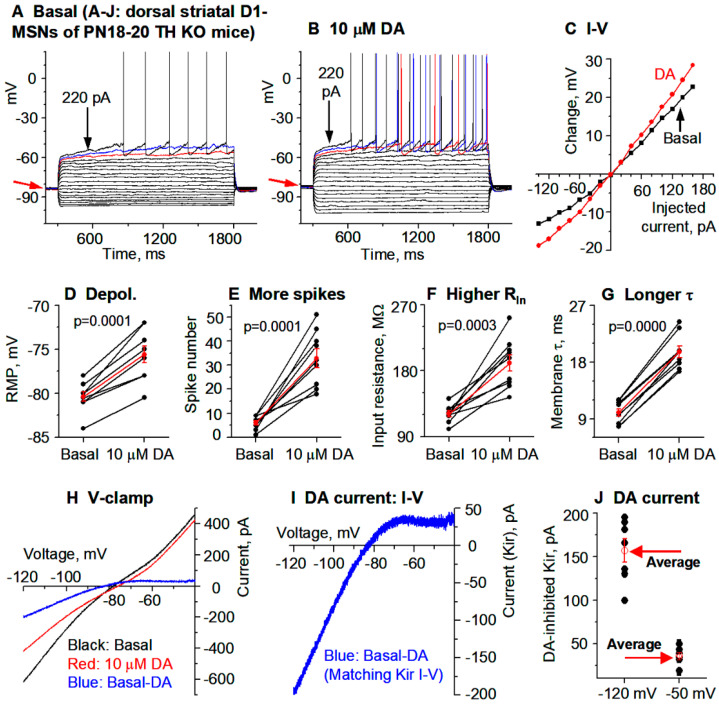
DA hyperactively increases D1-MSN excitability in the dorsal striatum (DS) in PN18-20 TH KO mice with total DA loss. (**A**–**C**) An example D1-MSN receiving hyperpolarizing and depolarizing current injection before (**A**) and during (**B**) bath application of 10 µM DA and the quantification of membrane potential changes (**C**). (**D**–**G**) Pooled data showing the DA effect on RMP (**D**), action potential firing (**E**), R_In_ (**F**), and τ (**G**) in 8 D1-MSNs. *p* values are from paired t-tests. (**H**,**I**) Example voltage ramp (150 mV/s)-induced K currents (recorded in 1 µM and 0 mM Ca) in an D1-MSN before (control black) and during 10 µM DA (red) (**H**); the difference caused by DA (blue) was extracted by subtraction (**I**) and I–V profile of this DA-sensitive current is typical of Kir. (**J**) Pooled data of the DA-sensitive Kir currents at −120 mV and −50 mV in 7 D1-MSNs with the mean ± se plotted in red indicated by the red arrows. Spikes are truncated for displaying subthreshold responses.

**Current clamp experiments.** To examine the potential effects of DA/D1 agonism’s effects on D1-MSN intrinsic excitability, we first made current-clamp recording in D1-MSNs in the dorsal striatum in TH KO mice. To avoid the potential confound of synaptic activity, GABA_A_ blocker picrotoxin and NMDA and non-NMDA receptor blockers AP5 and DNQX were added to the bathing solution in this experiment. To monitor membrane properties at different membrane potentials, determine whole cell input resistance (R_In_), and evoke spike firing, we injected a series of current pulses (starting at −140 pA and increasing +20 pA per step) into D1-MSNs. Upon hyperpolarizing current injection, these D1-MSNs showed an inward rectification typical of striatal MSNs (Figure 3A) [13]. Upon injection of strong depolarizing currents, action potentials were evoked in D1-MSNs when the membrane potential reached around −42 mV in TH KO mice (Figure 3A); the action potential peak commonly reached around +30 mV with a duration of about 2.0 ms at the base (recorded at +31 °C)—these characteristics are typical for MSNs.

After obtaining a stable baseline recording, we bath-applied 10 μM DA and detected a consistent depolarization in D1-MSNs, and this effect was larger in TH KO mice than in WT mice. As shown in Figure 3A–C, and Table 1, bath application of 10 μM DA caused a 4.8 ± 0.6 mV depolarization in DS D1-MSNs in TH KO mice, depolarizing them from their RMP at −80.4 ± 0.6 mV to −75.6 ± 1.0 mV under 10 μM DA (*p* = 0.00003, t = −8.63, DoF = 8, paired *t*-test) (Figure 3A,B,D; Table 1); simultaneously, bath application of 10 μM DA also increased the whole cell R_In_ from the basal 121.3 ± 3.9 MΩ to 190.4 ± 11.3 MΩ under 10 μM DA (measured by a −20 pA current from the RMP), a 57.2% increase (*p* = 0.0003, t = −6.05, DoF = 8, paired t-test) (Figure 3F; Table 1); the membrane τ was also increased from the basal 10.0 ± 0.6 ms to 19.6 ± 0.9 ms under 10 μM DA (*p* = 0.0000, t = −17.6, DoF = 8, paired t-test) (Figure 3G; Table 1)—this is expected because the membrane τ is proportional to whole cell R_In_. The increase in the whole cell R_In_ became larger when the cell membrane potential was more hyperpolarized (Figure 3C), indicative of a potential inhibition of an inwardly rectifying current or Kir (this will be confirmed by voltage clamp experiments described below).

Because of the DA-induced increase in R_In_, DA increased the current-injection-induced depolarization and the evoked spikes but did not change the membrane potential threshold for action potential triggering or waveform. Under the basal condition, ≥180 pA was often needed to evoke spikes (Figure 3A); during 10 μM DA, 140 pA was often sufficient to evoke spikes, such that 180 pA evoked many more spikes than under the control condition (Figure 3B,E): 6.0 ± 0.9 spikes were evoked by injected current pulses up to 180 pA under control, whereas 32.9 ± 3.9 spikes were evoked by the same current pulses during 10 μM DA (*p* = 0.0001, t = −7.06, DoF = 8, paired t-test) (Figure 3A,B,E; Table 1).

These DA excitatory effects (RMP depolarization, increases in R_In_ and membrane τ in DS D1-MSNs) in DD mice were also larger than those in WT mice (evident in the listed numbers in Table 1 and also supported by ANOVA testing).

**Voltage clamp experiments.** The voltage response profile in our current clamp data shown above suggest that DA may be inhibiting Kir in D1-MSNs (Figure 3A–C). To further establish this conclusion, here we performed voltage clamp experiments to directly examine DA’s potential effects on the Kir in D1-MSNs in TH KO mice. The voltage dependence or the I–V relationship of the DA-sensitive current was investigated by using a linear voltage ramp ranging from −120 mV to −40 mV. The 10 µM DA-sensitive current was extracted by subtraction. As shown in Figure 3H,I, the 10 µM DA-sensitive current had the characteristics of Kir: it was inwardly rectifying and had a reversal potential near the calculated K reversal potential at −87.5 mV for our extracellular and intracellular solution pair. The amplitude of the DA-sensitive Kir was −156.7 ± 13.5 pA at −120 mV and 36.0 ± 3.7 pA at −50 mV in 7 D1-MSNs in dorsal striatum (Figure 3J, Table 1). These characteristics strongly resemble the Kir typically seen in MSNs [14,15].

**Figure 4:**
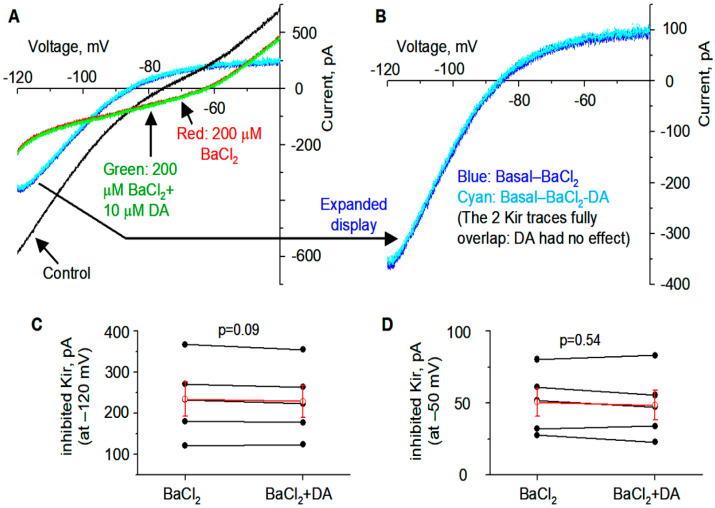
Ba inhibits Kir and occludes DA inhibition of Kir in D1-MSN in DS in PN18-20 TH KO mice. (**A**,**B**) Example voltage ramp (150 mV/s)-induced K currents (recorded in 1 μM TTX and 0 mM Ca) in a D1-MSN before (control black) and during 200 µM BaCl_2_ (red) and during 200 µM BaCl_2_ with 10 μM DA (green) (**A**); differences caused by BaCl_2_ (blue) and by BaCl2 with DA (cyan) were extracted by subtraction (**B**) and I–V profiles of this Ba/Ba+DA-sensitive current are typical of Kir. (**C**) Pooled data of the DA-sensitive Kir currents at −120 mV during 200 µM BaCl_2_ and during 200 μM BaCl_2_ with10 μM DA. (**D**) Pooled data of the DA -sensitive Kir currents at −50 mV during 200 μM BaCl_2_ and during 200 μM BaCl_2_ with10 μM DA. Red symbols and lines are the mean ± se.

Additionally, DA (10 µM) was without effect on the voltage ramp-evoked currents in the presence of 200 µM BaCl_2_, a known Kir blocker [13,15,16,51], further confirming that the DA-sensitive current was Kir (Figure 4) and also indicating that Kir was the dominant outward or K current inhibited by D1R activation. It is clear that 200 µM BaCl_2_ produced a total or nearly total inhibition of Kir, thus occluding the partial Kir inhibition normally produced by DA/D1R agonism.

### 3.3. DA Agonism Hyperactively Increases D1-MSN Excitability in the NAc in TH KO Mice with Total DA Loss

Like the DS, NAc receives intense DA innervation, and NAc DA activity has many important functions: motivation, reward, emotion, and limbic functions. Thus, understanding the cellular and ion channel mechanisms of DA regulation and dysregulation of NAc neurons is important. Like the DS, the effects DA on MSNs in the NAc were also controversial [7,52]. Here, we addressed this long-standing question. We focused on NAc core identified by using the anterior commissure as the landmark.

**Figure 5:**
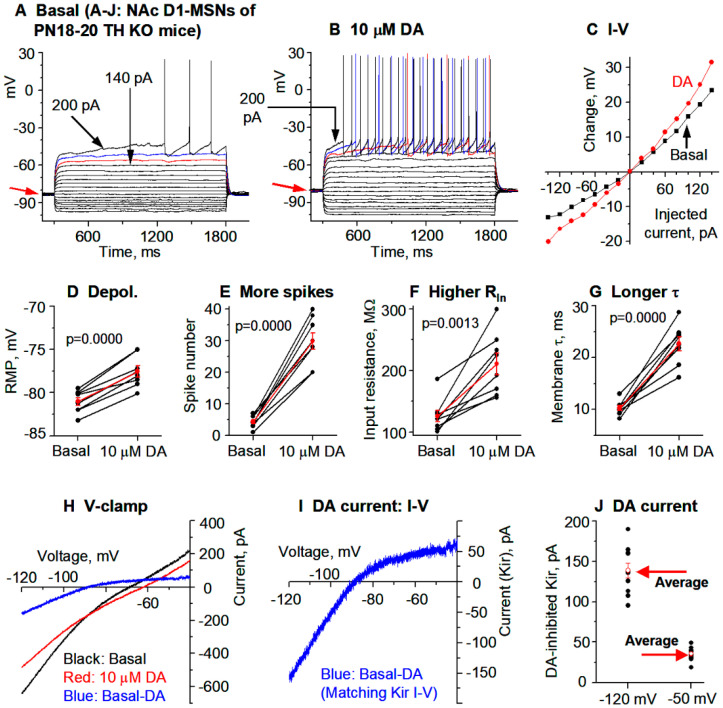
DA hyperactively increases D1-MSN excitability in the nucleus accumbens (NAc) in PN18-20 TH KO mice with total DA loss. (**A**–**C**) An example D1-MSN receiving hyperpolarizing and depolarizing current injection before (**A**) and during (**B**) bath application of 10 μM DA and the quantification of membrane potential changes (**C**). (**D**–**G**) Pooled data showing the DA effect on RMP (**D**), action potential firing (**E**), R_In_ (**F**), and τ (**G**) in 8 D1-MSNs. *p* values are from paired t-tests. (**H**,**I**) Example voltage ramp (150 mV/s)-induced K currents (recorded in 1 μM and 0 mM Ca) in an D1-MSN before (control black) and during 10 μM DA (red) (**H**); the difference caused by DA (blue) was extracted by subtraction (**I**) and I–V profile of this DA-sensitive current is typical of Kir. (**J**) Pooled data of the DA-sensitive Kir currents at −120 mV and −50 mV in 10 D1-MSNs with the mean ± se plotted in red indicated by the red arrows. Spikes are truncated for displaying subthreshold responses.

**Current clamp experiments.** Like D1-MSNs in the DS, upon bath application of 10 μM DA, the RMP was modestly but consistently depolarized by 3.5 ± 0.4 mV, from −81.0 ± 0.5 mV under control conditions to −77.5 ± 0.6 mV under 10 μM DA (*p* = 0.00003, t = −9.499, DoF = 7, n = 8 cells, paired *t*-test) (Figure 5A–C), the evoked spike numbers were increased from 4.4 ± 0.7 spikes under control to 29.9 ± 2.7 under 10 μM DA (n = 8 cells, *p* = 0.00002, t = −10.5045, DoF = 7, paired *t*-test) (Figure 5E; Table 1), and the whole cell R_In_ was increased from 125.9 ± 9.5 MΩ under basal condition to 210.8 ± 17.7 MΩ under 10 μM DA (n = 8 cells, *p* = 0.0007, t = −5.1586, DoF = 7, paired *t*-test) (Figure 5F; Table 1); additionally, likely as a consequence of increased whole cell R_In_, the membrane-charging time constant τ was also increased from 10.3 ± 0.5 ms during baseline to 22.5 ± 1.5 ms under 10 μM DA (n = 8 cells, *p* = 0.0007, t = −5.1586, DoF = 7, paired *t*-test) (Table 1). These effects were identical to those in DS (compare Figure 3 and Figure 5).

**Voltage clamp experiments.** Our current clamp data presented above strongly indicate that DA/D1R agonism may be inhibiting Kir in D1-MSNs in NAc in TH KO mice (Figure 5A–C). Here, we performed voltage clamp experiments to directly determine DA/D1R agonism’s potential effects on the Kir in NAc D1-MSNs in parkinsonian mice. We evoked Kir by using the same linear voltage ramp from −120 mV to −40 mV. As shown in Figure 5H,I, the 10 μM DA-sensitive/inhibited current, extracted by subtraction, was inwardly rectifying and had a reversal potential near the calculated K reversal potential at −87.5 mV; the amplitude of this DA-sensitive current was −138.7 ± 9.3 pA at −120 mV and 35.5 ± 2.7 pA at −50 mV in 10 D1-MSNs in NAc (Figure 5; Table 1). These results indicate that DA inhibits a current with characteristics of Kir in D1-MSNs in NAc in TH KO mice.

### 3.4. Hyperactive DA Responses in D1-MSNs Remain in More Mature TH KO Mice

**Figure 6:**
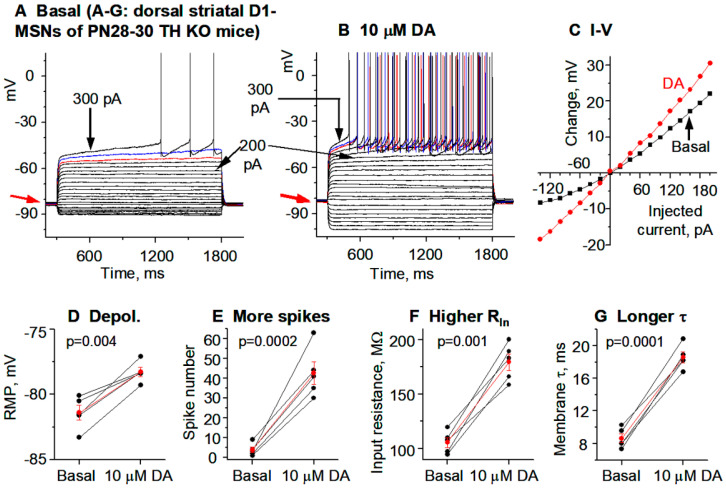
Hyperactive DA responses in D1-MSNs remain in the dorsal striatum in more mature PN28-30 TH KO mice. (**A**–**C**) An example D1-MSN receiving hyperpolarizing and depolarizing current injection before (**A**) and during (**B**) bath application of 10 μM DA and the quantification of membrane potential changes (**C**). (**D**–**G**) Pooled data showing the DA effect on RMP (**D**), action potential firing (**E**), R_In_ (**F**), and τ (**G**) in 5 D1-MSNs. Red symbols and lines are the mean ± se. *p* values are from paired t-tests. Spikes are truncated for displaying subthreshold responses.

We next examined DA effects in more mature PN28-30 mice. This is physiologically relevant and also because a recent study reported that DA deficiency increased D1-MSN intrinsic excitability around PN28 and thereafter but not at PN18 [24]. We found that the intrinsic membrane properties of D1-MSNs matured apparently in a similar or identical manner in DA innervation-intact mice and DA-denervated mice such that the baseline intrinsic membrane properties remained similar in DA innervation-intact mice and DA-denervated mice. Specifically, while there were some quantitative changes such as a decrease in whole cell R_In_ due to normal increased Kir expression as the animal becomes more mature, these parameters of the intrinsic membrane properties remained similar in D1-MSNs in DA innervation-intact mice and DA-denervated mice, and the hyperactive DA responses in D1-MSNs in DA-denervated striatum remained (Figure 6; Table 1).

### 3.5. Gradient Hyperactive DA Response in D1-MSNs in Pitx3Null Mice with Gradient DA Denervation

**Hyperactive DA enhancement of D1-MSN excitability in the dorsal striatum (DS) in Pitx3Null mice.** Pitx3Null mice have a gradient striatal DA denervation (Figure 1A,B) with the DA loss in the very dorsal DS being ~99% and the NAc DA loss being about 50% (weak to non-supersensitivity; hence, no DA receptor hyperfunctionality and no triggering of c-fos expression upon L-dopa stimulation [32]), resembling the DA loss pattern in PD patients and thus providing an outstanding opportunity for studying potentially gradient DA effects in parkinsonian striatum.

**Figure 7:**
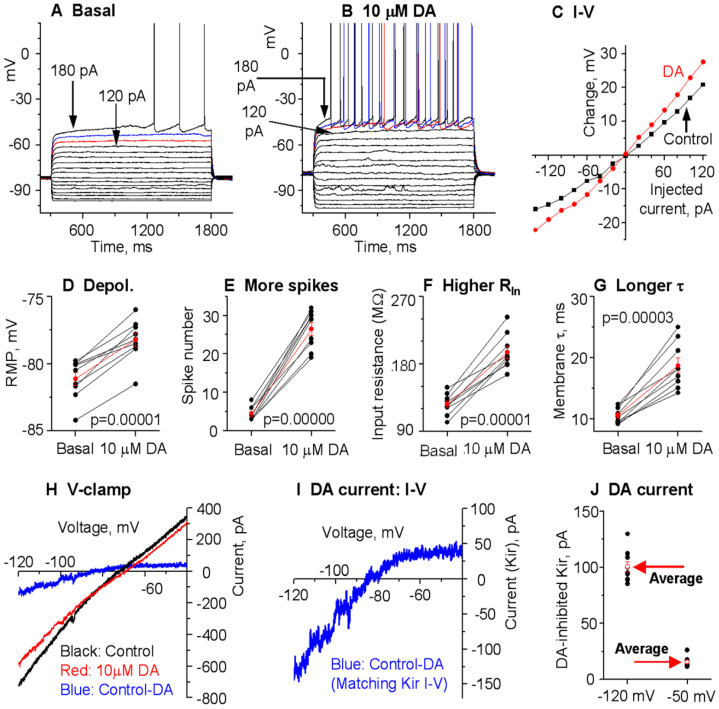
DA hyperactively increases D1-MSN excitability in the dorsal striatum (DS) in PN18-20 Pitx3Null mice with severe DA loss in DS. (**A**–**C**) An example D1-MSN receiving hyperpolarizing and depolarizing current injection before (**A**) and during (**B**) bath application of 10 µM DA and the quantification of membrane potential changes (**C**). (**D**–**G**) Pooled data showing the DA effect on RMP (**D**), action potential firing (**E**), R_In_ (**F**), and τ (**G**) in 9 D1-MSNs. *p* values are from paired t-tests. (**H**,**I**) Example voltage ramp (150 mV/s)-induced K currents (recorded in 1 µM and 0 mM Ca) in an D1-MSN before (control black) and during 10 µM DA (red) (**H**); the difference caused by DA (blue) was extracted by subtraction (**I**) and I–V profile of this DA-sensitive current is typical of Kir. (**J**) Pooled data of the DA-sensitive Kir currents at −120 mV and −50 mV in 9 D1-MSNs with the mean ± se plotted in red indicated by the red arrows. Spikes are truncated for displaying subthreshold responses.

We first used PN18-20 Pitx3Null mice and recorded D1-MSNs in the very dorsal part (within 300 µm from the corpus callosum) of DS where DA denervation is about 99% and D1Rs are hyperfunctional [32,38,53,54]. In current clamp, upon bath application of 10 μM DA, the RMP was depolarized from −81.1 ± 0.5 mV under control condition to −78.2 ± 0.5 mV under 10 µM DA (n = 9 cells, *p* = 0.00001, t = −9.4784, DoF = 8, paired *t*-test) (Figure 7A,B,D; Table 1), the evoked action potential numbers were increased from 4.4 ± 0.6 to 26.4 ± 1.7 (n = 9 cells, *p* = 0.00000, t = −14.758, DoF = 8, paired *t*-test) (Figure 7E; Table 1), the whole cell R_In_ was increased from 126.1 ± 4.7 MΩ under basal condition to 195.5 ± 7.9 MΩ under 10 µM DA (n = 9 cells, *p* = 0.00001, t = −9.3708, DoF = 8, paired *t*-test) (Figure 7F; Table 1), and, likely as a consequence of increased whole cell R_In_, the membrane-charging time constant τ increased from 10.6 ± 0.4 ms during baseline to 18.7 ± 1.4 ms under 10 μM DA (n = 9 cells, *p* = 0.00003, t = −8.3786, DoF = 8, paired *t*-test) (Figure 7G; Table 1).

We further performed voltage clamp experiments to directly determine DA’s potential effects on Kir in D1-MSNs in Pitx3Null mice. To evoke Kir currents, we used the same linear −120 mV to −40 mV voltage ramp. The 10 µM DA-sensitive current was extracted by subtraction. As shown in Figure 7, the 10 µM DA-inhibited current was inwardly rectifying and had a reversal potential near the calculated K reversal potential at −87.5 mV, matching the characteristics of Kir. The amplitude of the 10 μM DA-inhibited Kir was −100.3 ± 4.7 pA at −120 mV and −15.6 ± 1.5 pA at −50 mV in D1-MSNs in DS in Pitx3Null mice (Figure 7; Table 1).

**Figure 8:**
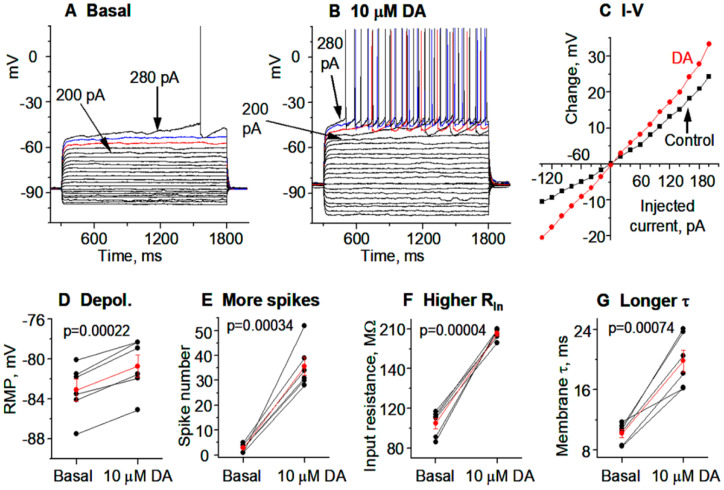
Hyperactive DA responses in D1-MSNs remain the dorsal striatum in more mature PN28-30 Pitx3 mice. (**A**–**C**) An example D1-MSN receiving hyperpolarizing and depolarizing current injection before (**A**) and during (**B**) bath application of 10 μM DA and the quantification of membrane potential changes (**C**). (**D**–**G**) Pooled data showing the DA effect on RMP (**D**), action potential firing (**E**), R_In_ (**F**), and τ (**G**) in 6 D1-MSNs. Red symbols and lines are the mean ± se. *p* values are from paired t-tests. Spikes are truncated for displaying subthreshold responses.

We next examined DA effects in more mature PN28-30 Pitx3Null mice. Data from more mature animals are physiologically important though more difficult to obtain. Additionally, a recent study reported that the DA deficiency in Pitx3Null mice affected the developmental maturation of D1-MSNs, such that D1-MSNs in PN28 or older Pitx3Null mice had more depolarized RMP and higher R_In_ than in WT mice, but these intrinsic excitability parameters were normal at PN18 [24]. We found that the basal RMP, R_In_, τ, and spike firing in D1-MSNs in PN28-30 Pitx3Null mice were similar to those in PN28-30 WT mice (Figure 8A–G). Furthermore, hyperactive DA responses in DS D1-MSNs remain in more mature Pitx3Null mice: 10 μM DA depolarized D1-MSNs in the DS in PN28-30 Pitx3Null mice and increased their R_In_, τ and evoked spike firing (Figure 8A–G).

**Figure 9:**
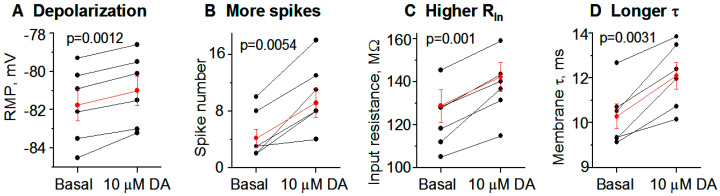
Largely normal DA response in Pitx3Null mouse NAc with moderate DA loss. (**A**–**D**) Pooled data showing the DA-induced depolarization of RMP (**A**) and effects on current-injection-induced action potential numbers (**B**), input resistance (**C**), and membrane τ (**D**) in 6 D1-MSNs. Red symbols and lines are the mean ± se. *p* values are from paired t-tests.

**Normal DA agonistic enhancement of D1-MSN excitability in the moderately DA-denervated NAc in Pitx3Null mice.** In PD, the ventral striatum or nucleus accumbens (NAc) retains significant amounts of DA innervation during middle PD and even in late-stage PD. How DA/D1R agonism affects the intrinsic excitability of NAc D1-MSNs with residual DA denervation in parkinsonian striatum was unknown. To address this question, we examined the effects of bath-applied DA on MSN intrinsic excitability of D1-MSNs in NAc in Pitx3Null mice where the residual DA innervation is significant (Figure 1A,B) and resembles the residual NAc DA innervation in middle-stage PD. Under a recording condition that was identical to that used for recording DS D1-MSNs, we detected, in current clamp recording mode, that bath application of 10 μM DA only modestly increased the intrinsic excitability of these NAc D1-MSNs in Pitx3Null mice (Figure 9, Table 1), just like the modest DA excitatory effect on D1-MSN intrinsic excitability in DA innervation-intact WT mice (Figure 1).

Based on the data described in preceding sections and ANOVA testing, it is clear that the baseline membrane properties (RMP, R_In_, τ, and evoked spike firing) of D1-MSNs were similar in the dorsal striatum and NAc in WT, Pitx3Null mice, and TH KO mice of mice, indicating that DA loss does not affect the development and maintenance of the membrane properties and the associated ion channels (especially the tonically active Kir, Kv channels, and Nav channels) of MSNs. In contrast, the DA-induced excitatory effects (depolarization, R_In_ increase, and τ increase) were larger in D1-MSNs in the DS and NAc in TH KO mice and the DS in Pitx3Null mice than in DS and NAc in WT mice and NAc in Pitx3Null mice, matching the pattern of DA loss severity, that is, DA’s excitatory effects on D1-MSNs were abnormally high or hyperactive in the striatal region where DA loss was severe.

### 3.6. Local Microinjection of BaCl2, a Kir Inhibitor, into the Dorsal Striatum Stimulates Movement and Occludes the Motor Stimulation of Microinjected D1 Agonist SKF81297

The data presented above indicate that D1 agonism excites D1-MSNs by inhibiting Kir; the literature data indicate that striatal D1 agonist microinjection stimulates movement [22] and that DA receptor-bypassing optogenetic activation of D1-MSNs is known to stimulate movement [11,12,21]. Thus, we reasoned that Kir may be a key ion channel mediating D1R agonistic stimulation of motor activity; hence, direct inhibition of Kir in D1-MSNs may stimulate movement and also occlude the motor stimulation of D1R agonism.

To test this idea, we used intra-striatal microinjection of BaCl_2_ and D1 agonist SKF81297. Ba^2+^ is a relatively selective Kir blocker with an IC_50_ around 10 μM [16,51,55], and 100–200 μM BaCl_2_ is often used to completely block Kir in MSNs [13,15,44]. We also used unilateral drug injection-induced unilateral rotations as the index of motor stimulation because (1) rotations can be reliably quantified and (2) bilateral drug injection often leads to bilaterally spatially asymmetric drug delivery, which interferes with the expression of motor activity and monitoring and quantification. Additionally, we focused on TH KO mice because (1) WT mice have plenty of endogenous DA that prevents the injected agonist from having any major effect and (2) with a total lack of endogenous DA, TH KO mice are likely to produce the strongest and hence most reliable responses to D1R agonism.

**Figure 10:**
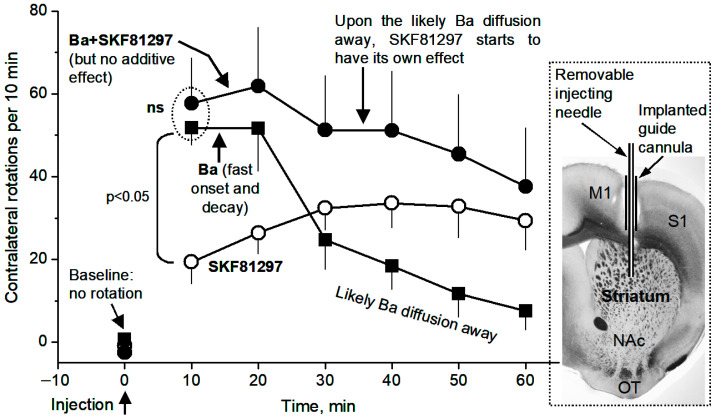
Inhibition of Kir occludes the motor stimulation of D1 agonism. SKF81297 (0.2 μg in 0.2 μL saline) unilateral microinjection into the DS induced contralateral rotations in 5 TH-KO mice, see Supplemental Video S1. BaCl_2_ (0.2 µg in 0.2 μL) unilateral microinjection into the DS induced robust contralateral rotations in 6 TH KO mice, see Supplemental Video S2. This Ba effect had a fast onset and also decayed quickly, likely because Ba can diffuse away quickly. Concurrent microinjection of BaCl_2_ (0.2 µg) and SKF81297 (0.2 µg in the same 0.2 µL together with BaCl_2_ in 7 TH KO mice) triggered only 58 rotations, similar to the 52 rotations triggered by BaCl_2_ microinjection alone. The data points at 10 min after injection are most pertinent to our question on Kir and motor stimulation and were analyzed with one-way ANOVA; data points at and after 30 min after drug injection likely involve differential diffusion of Ba and SKF81297 besides their respective pharmacological effects and thus were not analyzed statistically. The insert on the right shows our histological verification of the injection site. The chronically implanted guide cannula was 26 Ga (outer dia. 0.41 mm), and the injection needle was 33 Ga (outer dia. 0.18 mm) that was removed after injection for minimizing tissue damage; the guide cannula tip was above the intended injection site also for minimizing tissue damage. M1, primary motor cortex; S1, primary somatosensory cortex; NAc, nucleus accumbens; OT, olfactory tubercle.

As expected, unilateral intrastriatal microinjection of the D1 agonist SKF81297 (0.2 μg in 0.2 μL) induced robust motor stimulation in the form of contralateral rotations, in TH KO mice, reaching the peak effect at 30 min after injection and staying at the peak level (~30 rotations/10 min) for the next 30 min—the duration of our monitoring (Figure 10, Supplemental Video S1)—consistent with our prior studies in a different mouse model of DA deficiency [22].

Furthermore, intrastriatal BaCl_2_ microinjection (0.2 μg in 0.2 μL), in a separate experiment, also induced contralateral rotations that were qualitatively similar to SKF81297-induced contralateral rotations (Figure 10, Supplemental Video S2); the Ba effect also occurred faster, reaching the peak effect (~50 rotations/10 min) at 10–20 min after Ba injection, and decayed faster: the decay started at 20 min after Ba injection (Figure 10). The faster rise and decay are consistent with the fact that Ba is a simple divalent cation and can diffuse more easily, whereas SKF81297 is a much more complex molecule and likely diffuses and reaches the maximal sphere of influence more slowly than Ba.

Equally important, as shown in Figure 10, concurrent microinjection of BaCl_2_ (0.2 µg) and SKF81297 (0.2 µg in the same 0.2 µL together with BaCl_2_) did not produce an additive rotation-stimulating effect; in fact, during 10 min after microinjection, only 58 rotations were triggered, similar to the 52 rotations triggered by BaCl_2_ microinjection alone. This is not because the rotational motor capacity was saturated; these mice can make >100 rotations/10 min when stimulated strongly. Instead, these results indicate that Ba was blocking and then occluding the same target (i.e., Kir in D1-MSNs) that D1 agonism-cAMP also tried to inhibit. Further, Ba excites (via inhibiting Kir) both D1-MSNs (Figure 4) and D2-MSNs [15], and D2-MSN excitation is motor-inhibitory and counters the motor-stimulating effect of D1-MSN excitation; thus, Ba’s motor-stimulating effect via Kir inhibition in D1-MSNs would be even larger than what we observed here if the counter-effects of D2-MSNs were not there.

## 4. Discussion

**Figure 11:**
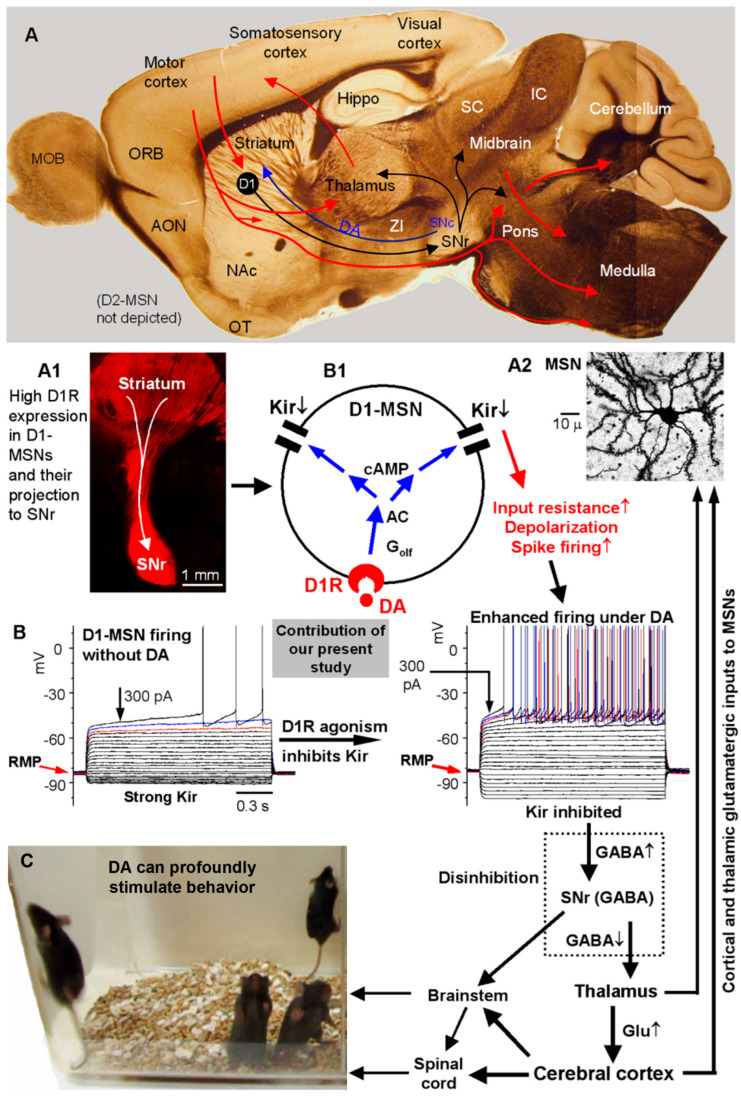
Integration of our present findings into the functional synaptic circuits of the cortico-basal ganglia-thalamo-cortical loop (**A**) that control motoric and cognitive behaviors. Striatal D1-MSNs (also known as dMSNs) selectively project to the GABAergic substantia nigra pars reticulata (SNr), a key output nucleus of the basal ganglia (**A1**). MSNs are so named because of their medium-sized cell body and numerous dendritic spines where synaptic inputs are received (**A2**). Our present findings fill a key knowledge gap. D1Rs are highly and selectively expressed in dMSNs—these neurons project precisely to SNr. D1R agonism inhibits the tonically active inwardly rectifying K current (Kir) and thus increases D1-MSN intrinsic excitability, spike firing (**B**,**B1**), and inhibitory synaptic GABA output, inhibiting GABA neurons in the SNr, disinhibiting the glutamatergic thalamus, and consequently promoting behavior (**C**); the 4 Pitx3Null male mice were treated with 10 mg/kg L-dopa and 5 mg/kg benserazide; 1 mg/kg D1 agonist SKF81297 had similar effects; similar motor stimulation was triggered in TH KO mice; no sex difference was observed). Not depicted here and not the topic of our paper, dopamine inhibits D2-MSNs, further stimulating behavior, i.e., D1 agonism and D2 agonism can stimulate behavior independently, but they normally work together synergistically [22]. Additionally, D1R activation is established to stimulate G_olf_ and then adenylyl cyclase (AC) and cAMP production. Our prior work using cAMP-producing Gs-GREADD also supports the mechanism depicted here [44]. Our present work fills an important knowledge gap about the key ion channel mediating the profound DA/D1 behavior stimulation. D2R agonism also stimulates behavior (synergistically with D1R agonism [22], unpublished data of Zhou lab). AON, anterior olfactory nucleus; MOB, main olfactory bulb; Glu, glutamate; Hippo, hippocampus; IC, inferior colliculus; SC, superior colliculus; NAc, nucleus accumbens; ORB, orbital cortical area; OT, olfactory tubercle; SNc, substantia nigra pars compacta; SNr, substantia nigra pars reticulata; ZI, zona incerta.

Our main findings are that (1) DA activation of D1Rs in D1-MSNs inhibits Kir, leading to substantially increased D1-MSN intrinsic excitability and spike firing, and this DA inhibition of Kir was occluded by BaCl_2_, (2) local BaCl_2_ microinjection-induced likely blockade of Kir stimulated motor activity and also occluded the motor stimulation of D1R agonism, indicating that Kir is a key mediator of DA's profound behavioral effects, and (3) DA loss does not affect the baseline intrinsic membrane properties but renders D1R agonistic enhancement of D1-MSN intrinsic excitability hyperactive. As illustrated in Figure 11, our results not only resolve the important and long-standing questions about how DA affects the intrinsic excitability and spike output of D1-MSNs, but also establish that inhibition of Kir in D1-MSNs is the key ion channel mechanism underlying D1R agonism's profound motor stimulation.

### 4.1. Hyperactive D1R Activation Inhibits Kir in D1-MSNs and Increases Their Intrinsic Excitability in Parkinsonian Animals Displaying Hyperactive D1R Agonistic Motor Response

Despite the intense expression of D1Rs and Kir in D1-MSNs, a D1R regulation of Kir and spike firing in D1-MSNs that can be logically interpreted in the context of D1-MSN function in the BG circuit and behavior had not been established until our present study. Employing the strategy of using DA-lacking TH KO mice that likely have larger cellular DA responses than normal mice, we collected robust data in DA-lacking mice that show unambiguously that the activation of cAMP-producing D1Rs inhibits Kir and increases the R_In_ in D1-MSNs in both the DS and NAc, thus substantially increasing the intrinsic excitability of these MSNs. The large amplitude and consistency of the DA excitation detected in both current clamp and voltage clamp strongly indicate that our results are reliable and correct and also make physiological sense as discussed below. In normal mice with intact striatal DA innervation in the striatum, DA induced excitatory effects in D1-MSNs qualitatively similar to those in parkinsonian mice, although these normal dopaminergic excitatory effects are much smaller.

Our present patch clamping results from in vitro brain slices are consistent with our published in vivo spike data and in vivo DA drug microinjection data, indicating that D1 agonism in the DA-denervated dorsal striatum triggers hyperactive spike firing in D1-MSNs and hyperactive motor activity [22,29]. Our present results are also consistent with Parker et al. (2018) [56] reporting that Ca-indicator-based multi-cell activity recording indicates that D1-MSNs responded hyperactively to L-dopa in 6-OHDA DA denervation PD mice, and with Ryan et al. (2018) [57] reporting that in vivo extracellular spike recording indicated that L-dopa overexcited D1-MSNs in 6-OHDA DA denervation PD mice. Thus, it is reasonable to conclude that DA/D1 agonism may directly increase D1-MSN spiking activity that in turn stimulates motor activity, although network effects may also contribute to the overall spiking activity following DA/D1 agonism administration. Our present data on DA effects on Kir obtained in brain slices are consistent with recent spike data recorded from awake parkinsonian animals that show that L-dopa or D1 agonism stimulated D1-MSN firing hyperactively in DA-depleted striatum [29,56,57,58].

The main D1R signaling pathway, in both normal and parkinsonian striatum, is to increase cAMP and activate the cAMP-PKA signaling pathway in D1-MSNs [59,60,61,62,63,64,65]. Thus, our results that D1R agonism and the likely consequent increase of intracellular cAMP inhibit Kir in D1-MSNs match our recent study that cAMP-producing Gs-DREADD activation inhibits Kir and increases intrinsic excitability in MSNs [44].

Another line of evidence supporting our data in this paper is the internal consistency in our data collected from D1-MSNs from WT mice, Pitx3Null mice, and TH KO mice; for example, due to the substantial residual DA innervation in the NAc in Pitx3Null mice, DA response is expected to be normal and similar to those in WT but different from those in DS in Pitx3Null and those in DS and NAc in TH KO mice. Our data show that this is indeed the case.

Prior studies examined DA regulation of D1-MSN intrinsic excitability in normal DA innervation-intact striatum, but the results were confounded. In WT mice, one study [66] detected a 60 µM DA-induced 30 pA inward current (the ion channel was not identified) in identified DS D1-MSNs but saw no change in input resistance; this result is difficult to interpret because at a fixed membrane potential, an inward current (or any current) requires a change in resistance, according to Ohm’s law. that study [66] further reported that 60 µM DA decreased the threshold current to trigger action potentials but increased the AP threshold potential (2 mV more depolarized); these opposing effects are difficult to interpret and potentially non-biological (i.e., they were potentially experimental artifacts): why would DA induce opposing effects on the same D1-MSNs? another study [67] reported that in normal NAc in mice, D1 agonism induced an inward current by blocking Kir, but the results were also confounded by the fact that the recordings were made in mixed, unidentified MSNs; consequently, in theory, 50% of the recorded cells should not respond to D1 agonist. A more recent study [10] reported that D1R agonism increased Kir in identified D1-MSNs and inhibited D1-MSN spike firing; but these results are not compatible with the overall function of D1Rs and the fact that D1-MSN activity stimulates movement [11] and D1 agonism in the striatum stimulates movements [22].

Our present results are not consistent with a recent paper [8] reporting substantially decreased Kir, increased input resistance, and more spike firing in D1-MSNs after 28 days of 6-OHDA-induced DA depletion. Although this inconsistency can only be resolved by future independent replication studies, we need to note two points here: first, this recent study [8] recorded Kir in the presence of 30 mM TEA, but it has been reported [13] that 25 mM TEA fully blocked the inward rectification or Kir in MSNs; second, the DA loss-induced D1-MSN high excitability recently reported [8] would lead to hyperkinesia, opposite to the fact that DA loss leads to hypokinesia. Additionally, our unpublished in vivo spike data indicate that D1-MSN spike firing is reduced, whereas D2-MSN firing is increased, in awake TH KO mice, supporting our brain slice patch clamp data here (Safa Bouabid and Fu-Ming Zhou, manuscript in preparation). Furthermore, D1R-phenocopying cAMP-increasing Gs-DREADD activation inhibits Kir and increases neuronal excitability [44].

We need to note here that in addition to D1R agonistic inhibition of Kir in D1-MSNs, D1R agonism may also inhibit depolarization-activated K currents and enhance inward currents such as the persistent Na current. These potential effects clearly can work together with Kir inhibition to amplify DA excitation of D1-MSNs and should be further studied. Indeed, it was reported [23] that a 5-pulse (20 Hz, mimicking burst firing) optogenetic stimulation (evoking DA release) increased D1-MSN excitability for “at least 10 min,” by inhibiting Kv and KCa currents, although replication studies are needed to confirm these findings because the DA axon terminal-released DA lasts only ~1 s [68,69].

### 4.2. DA Response Intensity in D1-MSNs Is Dependent on DA Denervation Severity

We found that in Pitx3Null mice, while DA had hyperactive excitatory effects on D1-MSNs in the dorsal striatum where DA denervation is very severe, the DA excitatory effects on the intrinsic excitability of D1-MSNs were modest and normal in the NAc where DA denervation is moderate and D1Rs are normal, as verified by a lack of L-dopa-induced c-fos expression [32]. This finding has two lines of importance. The first is that in PD, the DA denervation also has a dorsal–ventral gradient with the DA loss being the severest in the dorsal striatum and less severe in NAc throughout the stages of PD, especially during early and middle stages [70,71,72]. Our present neurophysiological data plus the c-fos data indicate that D1Rs in the central striatum and NAc are probably normal in early and middle stage PD, which may be a factor that L-dopa treatment induces few behavioral problems in these patients. In late stage PD when DA loss becomes severe even in VS, then D1Rs in central striatum and NAc also become abnormal and hyperfunctional, contributing to the dysregulation of brain functions such as cognition, emotion, and limbic functions. The second importance of our Pitx3 NAc data is that they provide an internal control (different DA loss severity leads to different D1R agonistic responses in a logical manner) and indicate the reliability of our data. This is important because of conflicting, probably incorrect data on this topic in the literature.

Our data from TH KO mice and Pitx3Null mice show that the nature of dopamine-depletion-induced cellular changes is similar in seemingly different animal models, i.e., TH-KO mice require daily L-dopa injection to survive, whereas Pitx3Null mice can survive without any treatment; but present data also show that the intensity and extension of dopamine-depletion-induced cellular changes are greater in TH-KO mice than in Pitx3Null mice (also note the dorsal vs. ventral response gradient in Pitx3Null mice) and are clearly dependent on the severity and extension of DA loss. This is consistent with our prior work that showed similar L-dopa-stimulated motor activity in mice with adult-onset 6-hydroxydopamine-induced symmetric dopamine denervation and in Pitx3null mice with perinatal-onset symmetric dopamine denervation, indicating that DA loss severity and pattern, not timing, are the key factors determining the molecular and motoric response intensity and pattern to L-dopa treatment [28]. Therefore, apparent differences in different dopamine depletion animal models are due to differences in dopamine loss severity and extension of dopamine loss, not intrinsic differences of different models. Because the dopamine-basal ganglia system in mammalian animals is remarkably conserved anatomically and functionally, knowledge obtained from dopamine depletion animal models can be used to understand the cell and molecular consequences of dopamine denervation and dopaminergic treatment in Parkinson’s disease.

### 4.3. D1-MSN Basal Intrinsic Membrane Excitability Is Slightly Low or Nearly Normal in Parkinsonian Striatum in a Brain Slice Preparation

A potential effect of DA loss on the intrinsic membrane properties such as RMP and R_In_ had been an unsettled important fundamental question with conflicting reports even for the same cell type in the same animal model. In brain slices from the Pitx3Null mice, one study [24] reported that D1-MSNs were hyperexcitable with a higher R_In_ and more depolarized RMP than in WT mice, but a second study [25] reported that, also in brain slices from Pitx3Null mice, D1-MSNs were hyperexcitable (but with a normal R_In_ and RMP) than in WT mice. Additionally, the second study [25] reported no change in D1-MSN R_In_ but reported a loss of dendrites and dendritic spines in Pitx3 mice; interpretation of this result is difficult because membrane loss due to dendrite loss should increase R_In_.

In our present study, we found that the basal intrinsic membrane properties and spike firing of D1-MSNs were similar in brain slices from WT mice, TH KO mice, and Pitx3Null mice, or the intrinsic excitability was only slightly lower in DA-depleted striatum. However, we need to note that DA release in WT mouse brain slices was severely reduced (compared to the in vivo condition) and dissipated quickly; thus, in intact animals, D1-MSN intrinsic excitability is probably significantly lower in DA-depleted striatum than in DA-intact striatum. Consequently, our present data contradict prior studies reporting increased D1-MSN excitability in 6-OHDA DA-depleted and Pitx3Null parkinsonian striatum in isolated brain slices [8,24,25,73]. These prior studies have a fundamental problem: an increased D1-MSN intrinsic excitability and spiking activity in parkinsonian striatum would increase motor activity (this chain of events is well established [11,12]), contradicting the fact that DA loss leads to akinesia or PD. In contrast, our data are fully consistent with the function of D1-MSNs in the normal striatum and the dysfunction of parkinsonian striatum: In normal striatum, the moderate D1R agonistic enhancement of D1-MSNs facilitates normal motor activity; loss of this D1R facilitation contributes to the reduced motor function in PD; in parkinsonian striatum, D1Rs are hyperfunctional, and, consequently, the D1R enhancement of D1-MSN excitability and hence the D1R facilitation of motor activity are also hyperfunctional, leading to motor function restoration and even motor hyperactivity depending on the dose of D1R agonism. Of course, D2Rs and D2-MSNs also contribute to the DA facilitation of motor function, but our focus here is D1Rs and D1-MSNs.

In contrast to the confounded literature data discussed above, our measured basal RMP and R_In_ of D1-MSNs from WT mice, Pitx3Null mice, and TH KO mice are consistent with and support each other. Our results are also consistent with in vivo intracellular recordings in MSNs showing that 6-OHDA-induced DA denervation did not alter the intrinsic membrane properties [74,75]. Further, these neurophysiological data are corroborated by our anatomical data that the MSN soma size, dendrite length, and dendritic spine number are similar in WT, Pitx3, and TH KO mice [32]; thus, the two factors that determine the MSN basal R_In_ are similar in normal and parkinsonian striatum: the anatomical structure (hence, membrane area) and the basal ion channel activity in the membrane.

### 4.4. Kir Is a Key Ion Channel Mediating DA/D1R Agonism’s Profound Motor Stimulation

In this study, we found that microinjection of the Kir blocker Ba into the DS triggers contralateral rotation and occludes the motor stimulation of D1R agonism, indicating that Kir is the common target for D1R agonism-induced intracellular signaling in D1-MSNs and direct Ba inhibition and that inhibition of Kir in D1-MSNs can stimulate movement. To our knowledge, this is the first identified, DA-regulated ion channel in BG or D1-MSNs that is directly connected to motor stimulation. Other ion channels such as sodium Nav channels may contribute to DA effects, but we failed to detect convincing evidence under our experimental conditions, in contrast to the strong evidence we have found for Kir in D1-MSNs. Additionally, although experimenter-induced Kir inhibition is sufficient to induce motor activity, natural DA/D1R-induced Kir inhibition likely occurs in coordination with multiple synaptic inputs (e.g., dopamine synaptic to activate D1R that in turn inhibits Kir and depolarizes D1-MSNs) and glutamatergic cortical inputs to directly depolarize MSNs—Kir inhibition-induced higher input resistance and depolarization can help cortical and thalamic glutamatergic synaptic inputs trigger spike firing and output from D1-MSNs, which promote motor and other behaviors. Thus, our present study reveals fundamental neurobiological mechanisms about Kir, its inhibition by D1R activation, and its direct behavioral function.

We also need to discussion this question: Because Ba inhibits Kir in both D1-MSNs and D2-MSNs non-selectively, how can Ba stimulate movement? We believe the following is the underlying mechanism. Although Ba inhibits Kir non-selectively, the behavioral consequences of Kir inhibition in D1-MSNs and D2-MSNs should be motor-promoting and motor-inhibiting, respectively, based on the results of optogenetic activation of D1-MSNs and D2-MSNs in the literature [11,12,21]. Thus, Ba-induced Kir inhibition in D1-MSNs in a unilateral striatum should stimulate motor function of the ipsilateral striatum and produce contralateral rotation; at the same time, Ba-induced Kir inhibition in D2-MSNs inhibits contralateral rotation. The fact that unilateral Ba injection into the striatum triggered contralateral rotation indicates clearly that Ba inhibition of Kir in D1-MSNs stimulates motor function, and this effect dominates; without the counter-effect in D2-MSNs, Ba’s stimulation of contralateral rotation, via Kir inhibition in D1-MSNs, probably would be larger than we observed here. Our data also indicate that the behavioral inhibitory effects of Ba excitation of D2-MSNs is overcome by the behavioral stimulating effects of Ba excitation of D1-MSNs. This logical interpretation is further supported by our unpublished data (Yuhan Wang and Fu-Ming Zhou) that unilateral microinjection of GABA_A_ receptor blocker bicuculline induced contralateral rotation despite bicuculline’s non-selective blockade of GABA_A_ receptors on all neurons and hence general excitation of all striatal neurons at and near the bicuculline injection site, also indicating that D1-MSN excitation-induced motor stimulation (here in the form of contralateral rotation) can overcome D2-MSN excitation-induced motor inhibition.

Microinjected Ba^2+^ can also inhibit Kir2 in striatal cholinergic interneurons (Kir2 expression is low in these neurons) and striatal GABA interneurons (Kir2 expression is also relatively low—that is a key reason MSNs have the most negative membrane potential). But motoric contribution from these interneurons is far smaller than the contributions from D1-MSNs and D2-MSNs, e.g., mice with their striatal cholinergic interneurons ablated can survive and have normal locomotor activity (with some abnormal behaviors) [76], and partial ablation of striatal GABA interneurons has only some very modest behavioral effects [77]; in contrast, MSN slow degeneration in Huntington’s disease causes profound motor and behavioral symptoms and eventually death of the patient [78]. These vast differences in the functional power of striatal MSNs vs. striatal interneurons are because MSNs comprise at least 90% of striatal neuronal population and are the output neurons of the striatum, whereas striatal interneurons comprise less than 10% of striatal neuronal population, are local interneurons, and exert their function by affecting MSNs. Therefore, striatal MSNs have the primary functional importance in the striatum, whereas striatal interneurons have a secondary functional importance. Thus, Ba effects on striatal interneurons (likely excitation of these neurons) are not likely to be the mediator of Ba-induced contralateral rotation, although a minor contribution is likely (the key mediator is Ba excitation of D1-MSNs, as discussed in the last paragraph).

Our microinjected BaCl_2_ (4 mM, 0.2 µL) was unlikely to have interfered with Ca-dependent processes such as glutamate release because, as indicated by the rapid onset and offset of the Ba stimulation of contralateral rotation, injected Ba likely diffused and diluted quickly such that it cannot significantly impede Ca influx. If Ba predominantly inhibits Ca influx and hence glutamate release, then D1-MSNs should be inhibited, and there should be no contralateral rotation, or ipsilateral rotation may be triggered, opposite to our observed contralateral rotation.

Additionally, for the following reasons, Kv channels are probably not the main target of our microinjected Ba^2+^. First, our data (Figure 4A,B) show that only in D1-MSNs, 200 µM BaCl_2_ inhibited Kir with no evidence of blocking Kv (though our voltage ramp ranged −120 mV to −40 mV, Kv is partially activated at −50 mV); we have published similar results for D2-MSNs: 200 µM BaCl_2_ inhibited Kir with no evidence of blocking Kv (using the same voltage ramp, −120 mV to −40 mV) [44]. Our data are consistent with the literature that at 200 µM, Ba^2+^ blocks Kir without affecting Kv [16,79,80]. Second, we injected nominally 0.2 µL 4 mM BaCl_2_: the actual amount injected into the tissue was probably less than 0.2 µL because it is a really very tiny volume, and some of 0.2 µL was probably lost in the injecting tubing; 0.2 µL was used to minimize tissue damage. It is safe to assume that once expelled from the needle tip, Ba^2+^ diffuses in all directions and thus gets diluted; the motor stimulation started quickly (peaked within 10 min after injection) and then also declined quickly (much more quickly than D1R agonist SKF81297); this timeline indicates that after diffusion and hence dilution, Ba^2+^ probably easily diluted to 500 µM−200 µM, but when Ba level declined to 100 µM and lower, Ba^2+^ can no longer strongly inhibit Kir in D1-MSNs, and thus motor stimulation started to decline. Kir is likely the main target because Ba^2+^ (200 µM in brain slice experiments like ours here) is widely used as a selective blocker of Kir (in MSNs and other brain neuron types), while TEA and 4-AP are wildly used to block Kv channels (with moderate selectivity), and Cs+ is often used to block all K channels. There are little data indicating efficient Ba2+ inhibition of mammalian neuronal Kv channels: we are only aware of this paper reporting external Ba inhibited cloned Kv2.1 with a Kd of 30 mM [81]. So, here, Kv contribution is minor.

Finally, Giβγ-activated Girk channels can be similarly blocked by Ba^2+^ [16,79,80], but Girk expression in MSNs is apparently low: we were unable to detect a DA-induced Girk current in D2-MSNs (neither in D1-MSNs), while we were able to reliably record a DA-induced Girk current in DA neurons; indeed, and Marcott et al. (2014) [82] used viral overexpression of Girk channels to detect a DA-induced Girk current (i.e., not native Girk current). In contrast, Kir expression in MSNs is high and widely reported in the literature [13]. So, Girk confounding was likely minimal. Taken together, Kir was probably the main target of microinjected BaCl_2_, and contributions from other K channels were probably minor.

## 5. Conclusions

In summary, our present study resolves two important long-standing questions. First, which is the most critical ion channel D1Rs regulate (inhibit or enhance) in D1-MSNs? Second, does this D1R regulation directly affect behavior? Now we have provided evidence showing that in D1-MSNs, DA activation of D1Rs in D1-MSNs inhibits the highly expressed and tonically active Kir and increases input resistance, intrinsic excitability, and spike firing, thus delineating an essential ion channel and cellular mechanism for the intense DA innervation and the heavily expressed D1Rs to regulate the striatum and promote behavior and cognition; in Parkinson’s disease, this mechanism is enhanced because of the hyperfunctional D1Rs (although this mechanism is off when DA treatment is off), enabling DA and D1R agonism to spectacularly stimulate behavior upon DA treatment (D2Rs and D2-MSNs also participate critically in DA promotion of behavior [22]).

## Figures and Tables

**Table 1:**
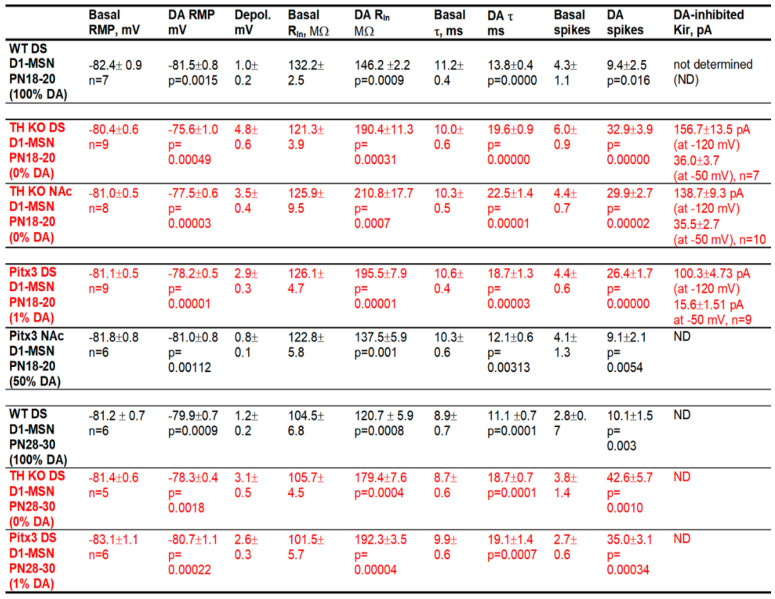
D1-MSN intrinsic membrane properties and their regulation by DA.

## Data Availability

The data presented in this study are available on request from the corresponding author.

## Supplementary Materials

The following supporting information can be downloaded at: https://www.mdpi.com/article/10.3390/brainsci15090979/s1, Video S1. SKF81297 (0.2 μg in 0.2 μL saline) microinjection into the dorsal striatum induced contralateral rotations; Video S2. BaCl2 (0.2 μg in 0.2 μL saline) microinjection into the dorsal striatum induced contralateral rotations.
