# Supplementary material for: Dopaminergic Inhibition of the Inwardly Rectifying Potassium Current in Direct Pathway Medium Spiny Neurons in Normal and Parkinsonian Striatum
Source: Brain Sci. 2025 Sep 12;15(9):979. doi: 10.3390/brainsci15090979 (PMC12468061; doi:10.3390/brainsci15090979)
Supplement: Supplementary file 1 [file brainsci-15-00979-s001.zip › Supl videos_2025-8-14.pdf]

Dopaminergic inhibition of the inwardly rectifying potassium current in direct pathway medium spiny neurons in normal and parkinsonian striatum

Qian Wang, Yuhan Wang, Francesca-Fang Liao, Fu-Ming Zhou*

Supplemental video data

Video 1. SKF81297 (0.2 μg in 0.2 μL saline) microinjection into the dorsal striatum induced contralateral rotations. This video clip was about 25 min after the start of injection. The microinjection tubing had been removed after the 5-min slow microinjection to avoid tissue damage and another 5 min for the injector to remain in place to avoid the reflux of the injected solution.

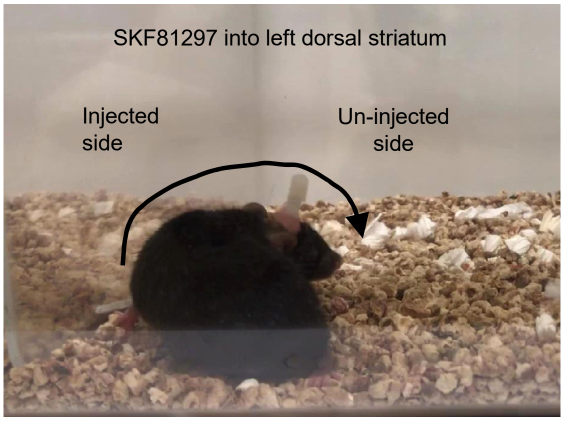

Video still from Video 1.

Video 2. BaCl2 (0.2 μg in 0.2 μL saline) microinjection into the dorsal striatum induced contralateral rotations. This video clip was about 8 min after the start of injection. The onset of BaCl2 effect was faster than that of SKF81297, see also Fig. 10. The microinjection tubing was still connected and visible because the microinjection cannula was allowed to remain 5 min after the 5-min slow microinjection to avoid tissue damage and the reflux of the injected solution.

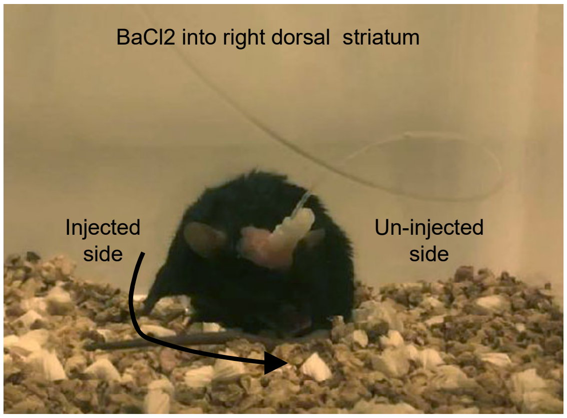

Video still from Video 2.
